# Incidência e Preditores de Desfechos Clínicos em Pacientes com Fibrilação Atrial Valvar e Não Valvar em uso de Antagonistas da Vitamina K

**DOI:** 10.36660/abc.20240147

**Published:** 2025-01-30

**Authors:** Idelzuita Leandro Liporace, Gustavo Bernardes F. Oliveira, Lucas Bassolli de Oliveira Alves, Nadia Marchiori Galassi, Andreia Dias Jeronimo, Fernanda Maria Lopes, Gregory Y. H. Lip, Álvaro Avezum

**Affiliations:** 1 Instituto Dante Pazzanese de Cardiologia São Paulo SP Brasil Instituto Dante Pazzanese de Cardiologia,São Paulo, SP – Brasil; 2 Hospital Alemão Oswaldo Cruz São Paulo SP Brasil Hospital Alemão Oswaldo Cruz, São Paulo, SP – Brasil; 3 University of Liverpool Liverpool Centre for Cardiovascular Science Liverpool Merseyside Reino Unido University of Liverpool – Liverpool Centre for Cardiovascular Science, Liverpool, Merseyside – Reino Unido; 4 Liverpool Heart & Chest Hospital Liverpool Reino Unido Liverpool Heart & Chest Hospital, Liverpool – Reino Unido; 5 Aalborg Universitet Aalborg Thrombosis Research Unit Aalborg Dinamarca Aalborg Universitet – Aalborg Thrombosis Research Unit, Aalborg – Dinamarca

**Keywords:** Acidente Vascular Cerebral, Tromboembolia, Hemorragia, Fibrilação Atrial, Varfarina

## Abstract

**Fundamento:**

Antagonistas da vitamina K (AVK) representam uma importante estratégia terapêutica oferecida pelo Sistema Único de Saúde no Brasil aos pacientes com fibrilação atrial (FA). Entretanto, os preditores de desfechos clínicos relevantes são pouco estudados no mundo real.

**Objetivo:**

Determinar a incidência e os preditores independentes de desfechos clínicos em pacientes com FA valvar e não valvar tratados com AVK.

**Métodos:**

Coorte prospectivo de pacientes com FA valvar e não valvar em uso ≥ 1 ano de AVK. Desfechos primários foram morte cardiovascular, eventos tromboembólicos, sangramento maior e não maior clinicamente relevante, separadamente e como desfecho composto, e adjudicados de modo independente. Valores de p < 0,05 foram considerados estatisticamente significantes.

**Resultados:**

Incluídos 1.350 pacientes, idade média de 69,2 (± 11.8) anos e 53,6% do sexo feminino, seguidos por 17 (15 - 19) meses. Incidência anual de eventos tromboembólicos e morte cardiovascular foi 4,4% e preditores foram tromboembolismo prévio ( *hazard ratio* [HR] 2,12; intervalo de confiança [IC] de 95% 1,22 - 3,67), tempo na faixa terapêutica (TFT) < 50% (HR 1,98; IC95% 1,16 - 3,37), e taxa de filtração glomerular (TFG) < 45 mL/min/1.73 m^2^ (HR 2,76; IC95% 4,82 - 1,58). Taxa de sangramento maior e não maior clinicamente relevante foram 3,24% por ano (IC95% 2,47 - 4,14) e preditores foram sangramento prévio (HR 2,60; IC95% 1,47 - 4,61) e prótese mecânica (HR 1,91; IC95% 1,15 - 3,15). O desfecho composto foi 8,7% por ano e preditores foram sangramento prévio (HR 1,70; IC95% 1,07 - 2,70), TFT < 41% (HR 1,79; IC95% 1,11 - 2,86) e diâmetro do átrio esquerdo > 44 mm (HR 1,97; IC95% 3,26 - 1,19).

**Conclusões:**

Tromboembolismo ou sangramento prévios, TFG e TFT reduzidos e átrio esquerdo aumentado foram preditores de desfechos clínicos em pacientes com FA tratados com AVK.

## Graphical abstract

**Figura Central:**
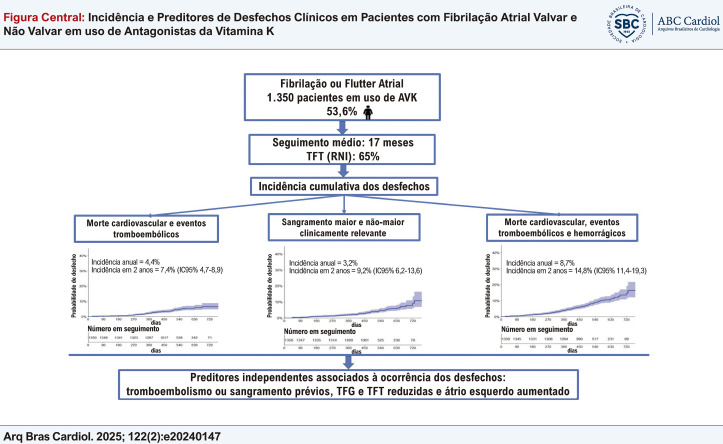
Incidência e Preditores de Desfechos Clínicos em Pacientes com Fibrilação Atrial Valvar e Não Valvar em uso de Antagonistas da Vitamina K

*AVK: antagonista da vitamina K; RNI: relação normalizada internacional; TFG: taxa de filtração glomerular; TFT: tempo na faixa terapêutica.*

## Introdução

A fibrilação atrial (FA) é a principal causa cardíaca de acidente vascular encefálico (AVE) tromboembólico, responsável por taxas elevadas de mortalidade, hospitalização, e incapacitação.^1 , 2^ Evitar o AVE tromboembólico é uma das prioridades no tratamento da FA e estudos da década de 90 revelaram que os antagonistas da vitamina K (AVK), em comparação com antiagregante plaquetário ou placebo, reduziram em média o risco de evento tromboembólico em 64% e de mortalidade em 26%.^3^ Embora o tratamento com AVK seja altamente eficaz na redução do risco de AVE, há dificuldade no manejo destes fármacos por diversos fatores.^4^

Nos últimos 15 anos, com o desenvolvimento dos anticoagulantes orais diretos (DOACs), houve uma nova perspectiva para o tratamento dos pacientes com FA. De fato, os grandes estudos pivotais com DOACs demonstraram a não inferioridade quando comparados ao AVK, sendo atualmente recomendados como a terapia anticoagulante de escolha para os pacientes com FA na ausência de estenose mitral reumática ou prótese mecânica.^5 , 6^

Entretanto, no Brasil, os DOACs não foram incorporados como terapia antitrombótica no Sistema Único de Saúde (SUS) devido ao custo.^7^ No nosso centro de anticoagulação, vinculado ao SUS, os pacientes que teriam indicação para uso de algum DOAC não apresentam condições para absorver os custos da medicação e permanecem recebendo anticoagulação com AVK.

Portanto, nosso estudo teve como objetivo identificar a incidência de desfechos clínicos relevantes e os preditores independentes de morte cardiovascular, eventos tromboembólicos, sangramento maior e não maior clinicamente relevante, em pacientes com FA valvar e não valvar tratados com AVK acompanhados em um setor de anticoagulação oral de um grande hospital terciário especializado em cardiologia.

## Métodos

Estudo de coorte prospectivo de pacientes com idade ≥ 18 anos, com FA ou flutter atrial valvar ou não valvar, tratados com AVK (varfarina ou femprocumona) por no mínimo 1 ano. Os pacientes foram recrutados de julho de 2017 a julho de 2018 e acompanhados de julho de 2017 a agosto de 2019, em São Paulo, Brasil. O único critério de exclusão foi gravidez. Conforme diretrizes vigentes na época da inclusão, os pacientes foram classificados em dois grupos: a) FA valvar: estenose valvar mitral moderada ou grave; próteses valvares mecânicas ou biológicas; ou história de procedimento de reparo/plastia valvar; e b) FA não valvar: demais casos sem algum dos critérios anteriores. As variáveis basais incluíram demografia, condição socioeconômica, fatores de risco cardiovascular, histórico médico relevante, exame físico, e testes laboratoriais. Os pacientes foram anticoagulados conforme titulação individual da dose semanal para faixas terapêuticas de relação normalizada internacional (RNI)-alvo: 2,5 a 3,5 (FA com próteses valvares mecânicas) ou 2 a 3 (demais pacientes com FA), conforme recomendações de diretrizes. O seguimento foi realizado mensalmente após a inclusão ou antecipado conforme julgamento clínico. O tempo na faixa terapêutica (TFT) foi calculado pelo método de Rosendaal.^8^ Os escores CHADS_2_,^9^ CHA_2_DS_2_-VASc,^10^ HAS-BLED^11^ e SAMe-TT_2_R_2_^12^ foram mensurados. Para o cálculo da taxa de filtração glomerular (TFG), utilizamos a fórmula de Chronic Kidney Disease Epidemiology Collaboration (CKD-EPI), e a disfunção renal foi classificada conforme National Kidney Foundation.^13^ O uso abusivo de álcool foi considerado como consumo de 8 ou mais doses semanais, conforme National Institute on Alcool Abuse and Alcholism.^14^ Utilizamos a classificação da International Society on Thrombosis and Haemostasis (ISTH), que define os sangramentos como maior (fatal, sintomático em área crítica ou órgão, causando uma queda no nível de hemoglobina de 2 g/dL ou mais, ou levando à transfusão de 2 ou mais unidades de sangue total ou concentrado de hemácias); não maior clinicamente relevante (sangramento agudo ou subagudo clinicamente evidente que não atende aos critérios para sangramento maior, mas desencadeia uma resposta clínica), e sangramento menor.^15^ As causas de óbitos foram adjudicadas por um observador independente, conforme Classificação Internacional de Doenças. Nos casos sem certidão de óbito disponível ou óbito ocorrido em domicílio ou em outro hospital, aplicou-se autópsia verbal para a determinação da causa da morte.^16^ Eventos cardiovasculares maiores, tromboembolismo, e sangramentos foram questionados em todas as consultas, classificados e registrados em formulários eletrônicos de acordo com a gravidade, valor do RNI no momento do evento, possíveis causas, e evolução. Os desfechos clínicos foram morte cardiovascular, eventos tromboembólicos, sangramento maior e não maior clinicamente relevante, separadamente e como desfecho composto.

Este é um estudo de iniciativa do pesquisador e foi aprovado pelo Comitê de Ética em Pesquisa da instituição sob o número CAAE: 68007417.5.40.5462. Todos os pacientes assinaram o termo de consentimento livre e esclarecido.

### Análise estatística

O cálculo do tamanho amostral foi realizado conforme estudos com AVK com TFT ≥ 65% como indicador adequado de anticoagulação. A estimativa de erro tipo I foi de 5% e o poder estatístico de 90%, resultando em amostra de 989 pacientes com FA para avaliar a qualidade da anticoagulação oral. A normalidade dos dados foi verificada pela inspeção de histogramas e aplicação do teste Shapiro-Wilk. As características dos grupos valvar e não valvar foram comparadas utilizando os testes qui-quadrado de Pearson, teste t de Student não-pareado ou Mann-Whitney, quando aplicável.

As variáveis contínuas foram expressas como média ± desvio-padrão ou mediana e intervalo interquartil (IQR) conforme normalidade dos dados e as variáveis categóricas foram descritas através de frequências absolutas (n) e relativas (%). A frequência de desfechos e eventos de interesse foi descrita em número absoluto, taxa de incidência, e taxa de incidência anualizada por 100 pessoas-ano. A incidência cumulativa dos desfechos durante o estudo foi estimada pelo método de Kaplan-Meier. O valor prognóstico dos potenciais preditores dos desfechos foi quantificado por razão de risco ( *hazard ratio* ), estimados por modelos de Cox. Análises multivariadas foram utilizadas para definir o conjunto de fatores de risco para a ocorrência dos desfechos, incluindo todas as variáveis selecionadas nas análises univariadas com nível de significância de 10% e variáveis com relevância clínica.^17^ O pressuposto de proporcionalidade dos riscos foi avaliado pela análise dos resíduos de Schoenfeld e então *hazard ratio* e intervalos de confiança de 95% derivados do método de Cox foram relatados. Os modelos finais foram ajustados por idade, sexo, presença de doença valvar, polifarmácia, e histórico de neoplasia por serem consideradas variáveis com relevância clínica para os desfechos. A acurácia discriminativa dos modelos finais foi avaliada pela área sob a curva ROC dos valores previstos pelos modelos (índice C). Os resultados foram apresentados para a população global e estratificados segundo o tipo de FA (valvar e não valvar). Os pontos de corte para variáveis contínuas foram estabelecidos por critérios clínicos ou por critérios estatísticos utilizando maxstat ( *maximally selected rank statistics* ).^18^ Dados ecocardiográficos faltantes foram imputados pelo método de imputação por equações encadeadas ou MICE, com base na idade, sexo, presença de doença valvar e uso de prótese mecânica.^19^ Todos os testes estatísticos foram bilaterais com valores de p < 0,05 denotando significância estatística. Todas as análises foram realizadas com o software estatístico RStudio 1.3.959.^20^

Modelos de riscos proporcionais de Cox foram utilizados para o ajuste multivariado da regressão logística para os desfechos estudados, e a estatística de Wald foi aplicada para o teste de hipóteses.

## Resultados

**Figura 1:**
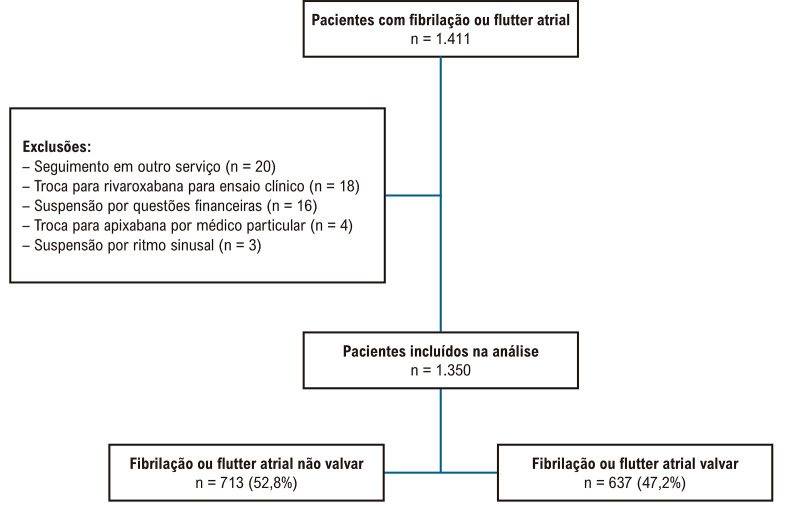
– Fluxograma do estudo.

Foram recrutados 1.411 pacientes com FA e, durante o seguimento, 61 foram excluídos, conforme apresentado na Figura 1 . Como descrição global, a média de idade foi 69,2 (± 11,8) anos e mulheres representando 53,6%. Varfarina representou 77,7% dos AVK. O tempo médio de uso de AVK antes da inclusão foi de 10,4 anos. Por definição clínica, 52,8% eram considerados com FA não valvar, 70% com FA permanente, e 8,5% com flutter atrial. A prevalência de comorbidades foi elevada entre os pacientes da amostra conforme apresentado na Tabela 1 .

**Tabela 1:** – Características clínicas do total de pacientes com fibrilação atrial e comparação entre os pacientes com fibrilação atrial não valvar e valvar

		Etiologia FA		
Variáveis	Amostra total (1.350)	Não valvar (n=713)	Valvar (n=637)	p
Idade, média (DP)	69,2 (±11,8)	73,7 (±10,0)	64,1 (±11,5)	< 0,001
Idade > 60 anos, n (%)	1.084 (79,5)	657 (92,1)	427 (67,0)	< 0,001
Sexo feminino, n (%)	724 (53,6)	314 (44,0)	410 (64,4)	< 0,001
Etnia branca/amarela, n (%)*	845 (62,7)	448 (62,9)	397 (62,5)	0,879
Escolaridade fundamental, n (%)*	980 (73,0)	557 (78,5)	517 (81,7)	0,141
Hipertensão arterial, n (%)	1.011 (74,9)	610 (85,6)	401 (63,0)	< 0,001
Dislipidemia, n (%)	761 (56,4)	495 (69,4)	266 (41,8)	< 0,001
Diabetes mellitus, n (%)	351 (26,0)	248 (34,8)	103 (16,2)	< 0,001
Doença arterial coronariana, n (%)	234 (17,3)	177 (24,8)	57 (8,9)	< 0,001
Função renal, n (%)*				< 0,001
TFG ≥ 90 mL/min/m^2^	231 (17,1)	68 (9,6)	163 (25,6)	
TFG 60 a 89 mL/min/m^2^	637 (47,2)	336 (47,2)	301 (47,3)	
TFG 45 a 59 mL/min/m^2^	277 (20,5)	175 (24,6)	102 (16,0)	
TFG 30 a 44 mL/min/m^2^	159 (11,8)	104 (14,6)	55 (8,6)	
TFG < 30 mL/min/m^2^	44 (3,3)	29 (4,1)	15 (2,4)	
Fração de ejeção, em %, mediana (q1-q3)	59 (18; 79)	60 (49-64)	58 (49-62)	0,297
Diâmetro AE, mediana (q1-q3)	49 (24; 126)	47 (42-52)	52 (48-58)	< 0,001
FA paroxística, n (%)	314 (23,3)	185 (25,9)	129 (20,3)	0,013
Flutter atrial	115 (8,5)	64 (9,0)	51 (8,0)	0,524
Tempo de ACO, mediana (q1-q3)	10,4 (7,7-14,7)	119 (97-154)	140 (73-215)	< 0,001
TFT Rosendaal < 65%	664 (49,2)	290 (40,7)	374 (58,7)	< 0,001
Antiagregação plaquetária, n (%)	131 (9,7)	90 (12,6)	41 (6,4)	< 0,001
Troca femprocumona-varfarina, n (%)	301 (22,3)	129 (18,1)	172 (27,0)	< 0,001
Polifarmácia, n (%)	789 (58,4)	484 (67,9)	305 (47,9)	< 0,001
Insuficiência cardíaca, n (%)	493 (36,5)	250 (35,1)	243 (38,1)	0,240
IMC ≥ 30 kg/m^2 *^	371 (27,5)	228 (32,0)	143 (22,4)	< 0,001
AVE isquêmico prévio, (%)	218 (16,1)	117 (16,4)	101 (15,9)	0,782
Tromboembolismo prévio, n (%)	263 (19,5)	132 (18,5)	131 (20,6)	0,342
Câncer, n (%)	32 (2,4)	25 (3,5)	7 (1,1)	0,004
Função hepática alterada, n (%)	22 (1,6)	13 (1,8)	9 (1,4)	0,552
Sangramento prévio, n (%)	164 (12,1)	79 (11,1)	85 (13,3)	0,204
CHADS_2_, mediana (q1-q3)	3,0 (2,0 – 4,0)	3,0 (2,0 - 4,0)	2,0 (1,0 - 3,0)	< 0,001
CHA_2_DS_2_-VASC, mediana (q1-q3)	3,0 (2,0 – 5,0)	4,0 (3,0 - 5,0)	3,0 (2,0 - 4,0)	< 0,001
HAS-BLED, mediana (q1-q3)	3,0 (2,0 – 3,0)	3,0 (3,0 - 4,0)	3,0 (2,0 - 3,0)	< 0,001
SAMe-TT_2_R_2_, mediana (q1-q3)	1,0 (1,0 – 3,0)	2,0 (1,0 - 3,0)	2,0 (1,0 - 3,0)	0,094
**Taxa de eventos**				
Desfecho composto: sangramento + evento TE, n (%)	118 (8,7)	57 (8,0)	61 (9,6)	0,304
Desfecho composto: evento TE + óbito CV (%)	60 (4,4)	31 (4,3)	29 (4,6)	0,855
Evento TE, n (%)	23 (1,7)	10 (1,4)	13 (2,0)	0,366
Desfecho composto: sangramento maior + NMCR, n (%)	62 (4,6)	26 (3,6)	36 (5,7)	0,079
Óbito CV	42 (3,1)	25 (3,5)	17 (2,7)	0,565

*ACO: anticoagulante; AE: átrio esquerdo; AVE: acidente vascular encefálico; CKD-EPI: Chronic Kidney Disease Epidemiology Collaboration; CV: cardiovascular; DP: desvio padrão; FA: fibrilação atrial; HR: hazard ratio; IMC: índice de massa corpórea; NMCR: não maior clinicamente relevante; q1-q3: intervalos interquartis; TFG: taxa de filtração glomerular; TFT: tempo na faixa terapêutica.*

### Resultados clínicos

Os pacientes foram acompanhados por mediana de 17 (IQR 15 a 19) meses. A mortalidade por todas as causas ocorreu em 6,2%, sendo 50% por causa cardiovascular, com uma taxa de sobrevivência global em dois anos de 90,5%. Eventos isquêmicos ou trombóticos ocorreram em 1,7%, com taxa anual de 1,18. Sangramentos maiores e não maiores clinicamente relevantes foram 4,6%, com taxa anual de 3,23, conforme Tabela 1 .

### Desfecho composto por morte cardiovascular ou evento tromboembólico

**Figura 2:**
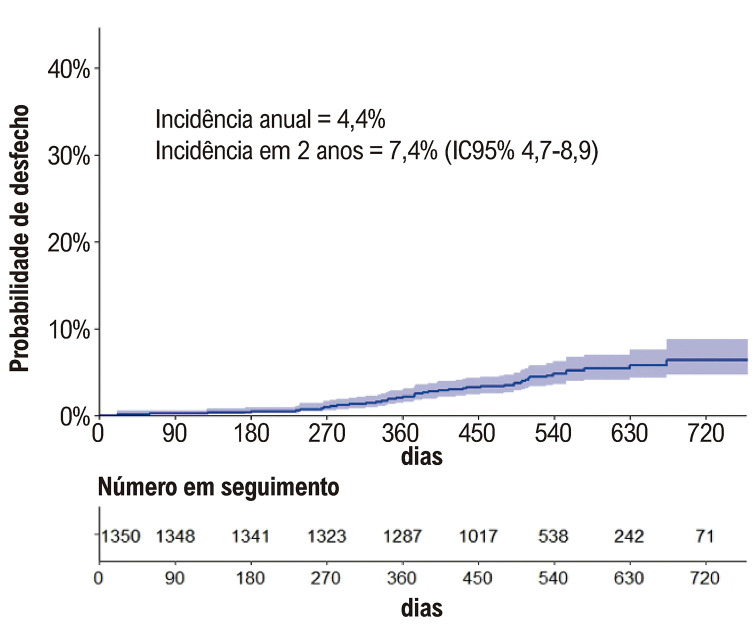
– Incidência cumulativa do desfecho composto por morte cardiovascular e eventos tromboembólicos.

A incidência cumulativa do desfecho foi 4,4% por ano e 7,4% em 2 anos ( Figura 2 ). A análise multivariada identificou tromboembolismo prévio, TFG < 45 mL/min/m^2^, TFT < 50% e diâmetro do átrio esquerdo (AE) como preditores independentemente associados à ocorrência do desfecho composto por morte cardiovascular ou evento tromboembólico, mostrado na Tabela 2 .

**Tabela 2:** – Análise multivariada de preditores associados aos desfechos clínicos tromboembólicos (A), hemorrágicos (B) e compostos por desfechos tromboembólicos e hemorrágicos (C) na população total com fibrilação atrial

**A**				
	*Hazard ratio* (IC95%) *	**p**	***Hazard ratio* (IC95%) ajustado ^ⱡ^**	**p**
TFT < 50%	1,98 (1,16-3,37)	0,013	1,95 (1,12-3,40)	0,019
TFG < 45 mL/min/m^2^	2,76 (1,58-4,82)	< 0,001	2,62 (1,43-4,82)	0,002
Tromboembolismo prévio	2,12 (1,22-3,67)	0,015	2,08 (1,21-3,61)	0,009
Diâmetro AE (contínuo)	1,03 (1,00-1,05)	0,015	1,03 (1,01-1,05)	0,009
* n = 1.348; índice C = 0,714 (± 0,03). AE: átrio esquerdo; IC: intervalo de confiança; TFG: taxa de filtração glomerular; TFT: tempo na faixa terapêutica. ^ⱡ^ Ajustado por: faixas etárias (60 a 80 anos, < 60 anos e > 80 anos), sexo, fibrilação atrial valvar, polifarmácia e neoplasia. Índice C = 0,717 (± 0,03).				
**B**				
	*Hazard ratio* (IC95%)*	**p**	***Hazard ratio* (IC95%) ajustado ^ⱡ^**	**p**
Prótese mecânica	1,91 (1,15-3,15)	0,012	1,98 (0,89-4,40)	0,094
Sangramento prévio	2,60 (1,47-4,61)	0,001	2,51 (1,41-4,47)	0,002
* n = 1.350; índice C = 0,634 (± 0,03). IC: intervalo de confiança. ^ⱡ^ Ajustado por: faixas etárias (60 a 80 anos, < 60 anos e > 80 anos) sexo, fibrilação atrial valvar, polifarmácia e câncer. Índice C = 0,649 (± 0,03).				
**C**				
	*Hazard ratio* (IC95%) *	**p**	***Hazard ratio* (IC95%) ajustado ^ⱡ^**	**p**
TFT Rosendaal < 41%	1,79 (1,11-2,86)	0,016	1,74 (1,08-2,82)	0,024
Átrio esquerdo > 44 mm	1,97 (1,19-3,26)	0,008	1,93 (1,14-3,24)	0,014
História de sangramento	1,70 (1,07-2,70)	0,026	1,66 (1,04-2,65)	0,033

* n = 1.350; índice C = 0,622 (± 0,02). IC: intervalo de confiança; TFT: tempo na faixa terapêutica. ^ⱡ^ Ajustado por: faixas etárias (60 a 80 anos, < 60 anos e > 80 anos) sexo, fibrilação atrial valvar, polifarmácia, câncer e Tromboembolismo prévio. n = 1.350; índice C = 0,638 (± 0,02). Modelos de riscos proporcionais de Cox foram utilizados para o ajuste multivariado de risco para os desfechos estudados e a estatística de Wald aplicada para o teste de hipóteses.

### Desfecho composto por sangramento maior e/ou não maior clinicamente relevante

**Figura 3:**
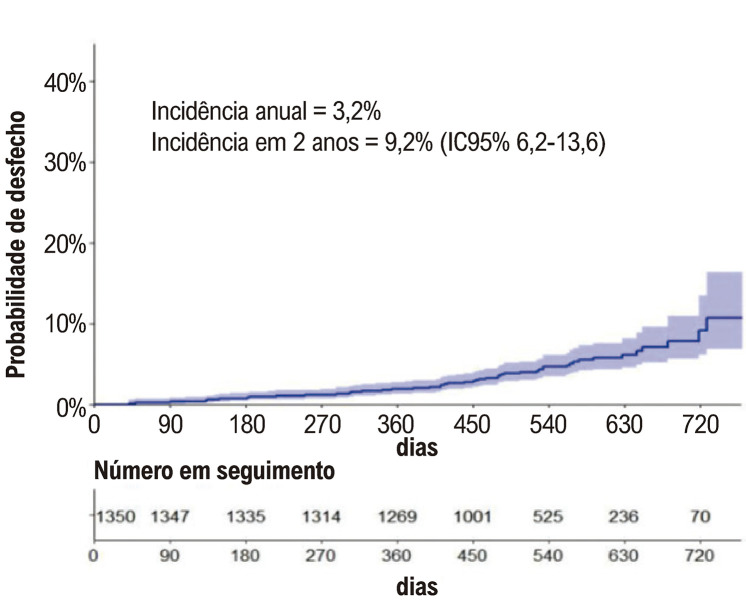
– Incidência cumulativa do desfecho composto por sangramento maior e não maior clinicamente relevante.

A taxa de sangramento anual foi 3,23%, e pacientes com prótese valvar mecânica e aqueles com sangramento prévio apresentaram maior risco de novos sangramentos ( Tabela 2 e Figura 3 ).

### Desfecho composto de morte cardiovascular, evento tromboembólico ou sangramento maior e/ou não maior clinicamente relevante

**Figura 4:**
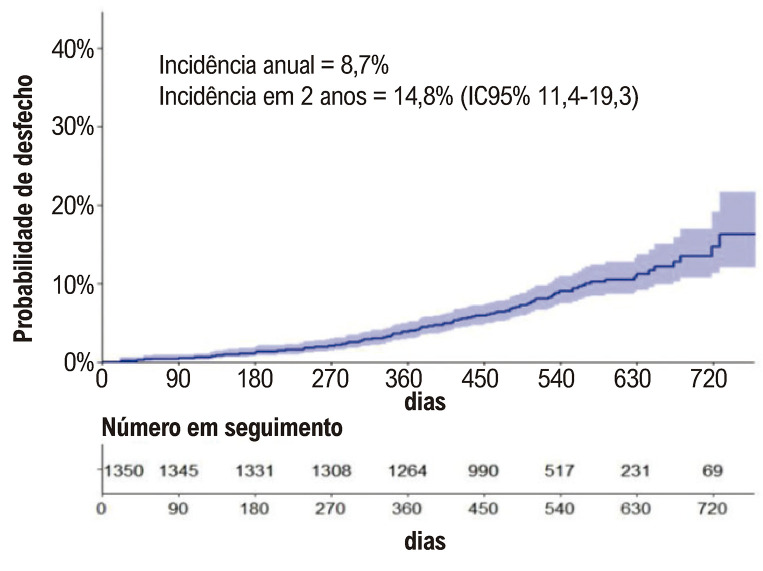
– Incidência cumulativa do desfecho composto por morte cardiovascular, eventos tromboembólicos e hemorrágicos.

A combinação de desfechos trombóticos e de sangramentos foi observada em 8,7%. A Figura 4 mostra a incidência cumulativa em 2 anos. Os preditores independentes foram sangramento prévio, TFT < 41% e diâmetro do AE > 44 mm, como apresentado na Tabela 2 .

### Comparação entre os pacientes com FA não valvar versus valvar

Do total da amostra, 52,8% foram classificados como FA não valvar e 47,2%, FA valvar ( Figura 1 ). As características dos pacientes com FA não valvar e valvar foram diferentes com significância estatística em vários aspectos, motivando a realização da análise dos desfechos de acordo com os dois grupos. A média de idade dos pacientes com FA não valvar foi 73,7 anos versus 64,1 anos naqueles com FA valvar. No momento da inclusão, 92,1% dos pacientes com FA não valvar tinham mais de 60 anos, em comparação com 67% no grupo FA valvar. As mulheres representaram 64,4% do grupo de FA valvar versus 44% dos pacientes com FA não valvar. Em relação à etnia e escolaridade autorreferidas, não foi observada diferença entre os grupos ( Tabela 1 ).

Pacientes com FA não valvar apresentaram maior prevalência de hipertensão arterial sistêmica, dislipidemia, diabetes mellitus, obesidade, doença arterial coronária, insuficiência renal grave, uso de antiplaquetários e polifarmácia. A apresentação permanente da FA também foi mais frequente entre aqueles com FA valvar, enquanto o tipo paroxístico foi mais prevalente na FA não valvar. Os escores de risco CHADS_2_, CHA_2_DS_2_-VASc e HAS-BLED foram mais elevados nos pacientes com FA não valvar versus FA valvar. A mediana do escore SAMe-TT_2_R_2_ foi semelhante para os dois grupos. O maior tempo de uso de anticoagulante e a frequência de pacientes com menor TFT (< 65%) foram mais comuns no grupo de FA valvar, mostrado na Tabela 1 .

Entre pacientes com FA não valvar, TFT < 50%, diâmetro do AE, e TFG < 45 mL/min/m^2^ ( *hazard ratio* 2.20; intervalo de confiança de 95% 1,01 a 4,77) foram preditores independentes para o desfecho composto de eventos isquêmicos e de sangramento. O TFT < 50%, diâmetro do AE e sangramento prévio foram preditores independentes para a ocorrência do desfecho composto de eventos trombóticos e hemorrágicos, conforme a Tabela 3 . Os desfechos isolados e compostos no grupo FA valvar também estão demonstrados na Tabela 4 .

**Tabela 3:** – Análise multivariada de preditores associados aos desfechos tromboembólicos (A) e composto por desfechos tromboembólicos e hemorrágicos (B) no grupo FA não valvar

**A**				
	*Hazard ratio* (IC95%) *		**p**	
TFT Rosendaal < 50%	4,12 (1,97-8,63)		< 0,001	
TFG < 45 mL/min/m^2^	2,20 (1,01-4,77)		0,046	
Diâmetro AE (contínuo)	1,05 (1,02-1,07)		< 0,001	
* n = 712; índice C = 0,755 (± 0,05). AE: átrio esquerdo; IC: intervalo de confiança; TFG: taxa de filtração glomerular; TFT: tempo na faixa terapêutica. odelo estratificado por idade ≥ 84 anos. Esta variável embora significativa violou o pressuposto de proporcionalidade dos riscos.				
**B**				
	*Hazard ratio* (IC95%)*	**p**	***Hazard ratio* (IC95%) ajustado ^ⱡ^**	**p**
TFT Rosendaal < 50%	2,54 (1,42-4,54)	0,002	2,64 (1,47-4,76)	0,001
Sangramento prévio	2,25 (1,21-4,17)	0,010	2,18 (1,17-4,07)	0,015
Diâmetro AE (contínuo)	1,03 (1,01-1,05)	0,001	1,04 (1,02-1,06)	0,001

* n = 713; índice C = 0,673 (± 0,04). AE: átrio esquerdo; IC: intervalo de confiança; TFT: tempo na faixa terapêutica. ^ⱡ^ Ajustado por: idade, sexo, polifarmácia e neoplasia. Índice C = 0,671 (± 0,04). Modelos de riscos proporcionais de Cox foram utilizados para o ajuste multivariado de risco para os desfechos estudados e a estatística de Wald aplicada para o teste de hipóteses.

**Tabela 4:** – Análise multivariada de preditores associados aos desfechos tromboembólicos (A) e composto por desfechos tromboembólicos e hemorrágicos (B) no grupo FA valvar

**A**				
	*Hazard ratio* (IC95%) *		**p**	
Idade ≥ 59 anos	0,38 (0,18-0,81)		0,013	
TFG < 45 mL/min/m^2^	2,84 (1,11-7,25)		0,029	
* n = 636; índice C = 0,629 (± 0,05). IC: intervalo de confiança; TFG: taxa de filtração glomerular.				
**B**				
	*Hazard ratio* (IC95%)*	**p**	***Hazard ratio* (IC95%) ajustado ^ⱡ^**	**p**
TFT Rosendaal < 38%	2,08 (1,10-3,92)	0,024	1,96 (1,02-3,74)	0,042
Tromboembolismo prévio	1,73 (1,00-3,01)	0,052	1,78 (1,02-3,11)	0,044
Diabetes mellitus	1,83 (1,03-3,25)	0,040	1,71 (0,93-3,13)	0,083
TFG < 45 mL/min/m^2^	1,93 (1,00-3,73)	0,050	2,09 (1,04-4,19)	0,039

* n = 636; índice C = 0,649 (± 0,03). IC: intervalo de confiança; TFG: taxa de filtração glomerular; TFT: tempo na faixa terapêutica. ^ⱡ^ Ajustado por: idade, sexo, polifarmácia e neoplasia. Índice C = 0.659 (± 0.03). Modelos de riscos proporcionais de Cox foram utilizados para o ajuste multivariado de risco para os desfechos estudados e a estatística de Wald aplicada para o teste de hipóteses.

## Discussão

Até o momento da submissão deste manuscrito, ao nosso conhecimento, esta representa a maior coorte prospectiva brasileira derivada do mundo real com experiência no uso de AVK em pacientes com FA no amplo espectro. De fato, avaliamos os preditores de desfechos clínicos relevantes, ou seja, eventos trombóticos, sangramentos, e óbitos em pacientes com FA ou flutter atrial considerados como valvar ou não valvar, atendidos em um hospital público, o que reflete a prática clínica mais comum nessa condição. Ressaltamos as características demográficas de idade avançada, predomínio de mulheres, e baixo nível econômico e de escolaridade.

Durante o seguimento de 1.950 pessoas-ano, observamos uma prevalência elevada de comorbidades, principalmente hipertensão arterial sistêmica, insuficiência cardíaca, diabetes mellitus, obesidade e disfunção renal, correspondendo a fatores de risco relacionados ao desenvolvimento de FA.^21^ A mediana de TFT observada em nossa amostra foi de 65%, menor do que valores observados na Espanha (70,3%) e Alemanha (81,4%),^22^ mas semelhante à taxa encontrada nos Estados Unidos e Canadá (64,1%)^23^ e maior do que revelado na Lituânia (40%)^24^ e em países africanos (30,8%).^25^

### Desfecho composto por morte cardiovascular ou evento tromboembólico

A ocorrência do desfecho composto por morte cardiovascular ou evento tromboembólico foi observada em 4,4%, semelhante à taxa anualizada do desfecho de eficácia encontrada naqueles em uso de varfarina do estudo randomizado *Edoxaban versus Warfarin in patients with Atrial Fibrillation* (ENGAGE-AF), que foi de 4,43%.^26^ Entretanto, foi discretamente superior à taxa de 3,51% no ensaio clínico *Dabigatran versus warfarin in patients with atrial fibrillation* (RE-LY) e à de 2,2% nos pacientes do estudo *Rivaroxaban versus warfarin in nonvalvular atrial fibrillation* (ROCKET-AF).^27 , 28^ Essa diferença pode estar relacionada à presença de pacientes com maior gravidade clínica, portadores de próteses mecânicas e estenose mitral moderada a grave na nossa amostra. A taxa de mortalidade total durante o seguimento do estudo foi 6,2%, sendo maior do que as encontradas no grupo varfarina nos grandes estudos pivotais com DOACs. No ensaio clínico RE-LY, a taxa de mortalidade total foi de 4,13%, no ROCKET-AF de 4,9%, e no *Apixaban versus warfarin in patients with atrial fibrillation* (ARISTOTLE) de 3,94%.^29^ Em relação à mortalidade cardiovascular, observamos 3,1%, semelhante à do ensaio clínico ENGAGE-AF que foi de 3,17%, porém, pouco acima da observada no estudo ARISTOTLE, de 2,02%.^26 , 29^ Nossa hipótese para os resultados encontrados é baseada na gravidade dos pacientes atendidos em nosso centro de anticoagulação, com grande volume de casos referenciados em fase mais avançada da cardiopatia, portadores de próteses mecânicas ou estenose mitral moderada a grave, diferentemente daqueles selecionados para ensaios clínicos randomizados. A incidência anual de eventos tromboembólicos foi 1,18%, sendo menor do que as observadas no grupo varfarina dos estudos RE-LY de 1,69%, ROCKET-AF de 2,2%, ARISTOTLE de 1,27% e ENGAGE-AF de 1,5%, como também no estudo que comparou varfarina com ácido acetilsalicílico ou placebo, com 1,4% de eventos tromboembólicos.^26 - 30^

Os fatores de risco independentes para a ocorrência do desfecho composto por morte cardiovascular ou eventos tromboembólicos foram tromboembolismo prévio, TFT < 50%, e TFG < 45 mL/kg/min. Resultados semelhantes foram observados no *Global Anticoagulant Registry in the FIELD–Atrial Fibrillation* (GARFIELD-AF), em que AVE isquêmico ou ataque isquêmico transitório prévios também foram associados a risco significativamente maior de mortalidade total. No mesmo registro, pacientes em uso de varfarina com TFT < 65% apresentaram risco 2,6 vezes maior de AVE isquêmico e 2,4 vezes maior de mortalidade total, em comparação com pacientes com níveis de TFT considerados adequados.^31^ Em um estudo publicado por Jones et al., uma redução de 10% no TFT esteve associada ao aumento de 29% no risco de mortalidade e de 10% a 12% em eventos tromboembólicos, incluindo AVE isquêmico,^32^ em consonância com nossos resultados.

### Desfecho composto por sangramento maior e/ou não maior clinicamente relevante

Em relação ao desfecho composto por sangramento maior e/ou não maior clinicamente relevante, de acordo com os critérios da ISTH, observamos incidência anual de 3,24%,menor que a demonstrada no grupo varfarina dos estudos ENGAGE-AF e ROCKET-AF, que apresentaram taxas de 13,02% e 14,5%, respectivamente.^26 , 28^ Os achados do presente estudo foram semelhantes às taxas encontradas em revisão sistemática de estudos randomizados de varfarina em comparação com aqueles sem anticoagulante, com frequência média anual de 3% em sangramento maior, e 9,6% em sangramentos maiores e menores.^26^

Os preditores associados à ocorrência do desfecho composto de sangramentos foram presença de próteses valvares mecânicas e sangramento prévio. Esse resultado foi semelhante ao descrito por Priksri et al., onde a presença de prótese mecânica em posição mitral foi fator de risco independente para a ocorrência de sangramento relacionado à varfarina.^33^

A taxa anual de sangramento maior isoladamente neste estudo foi de 1,29%, inferior às observadas nos grupos em uso de varfarina que foram de 3,4%, 3,09% e 3,43% nos estudos ROCKET-AF, ARISTOTLE e ENGAGE-AF, respectivamente.^26 , 28 , 29^

Neste estudo, a disfunção renal grave esteve associada à ocorrência de eventos tromboembólicos, mas não se associou ao aumento de sangramento maior ou clinicamente relevante, ao contrário do observado em outros estudos.^34^ Como exemplo, Lip et al. avaliaram uma coorte de 7.329 pacientes com FA dos estudos *Stroke Prevention Using an ORal Thrombin Inhibitor in Atrial Fibrillation* (SPORTIF III e V), que compararam o uso de varfarina com ximelagatrana, e a presença de insuficiência renal (TFG < 50mL/min/m^2^) foi um dos fatores associados à ocorrência de sangramento.^35^

### Desfecho composto de morte cardiovascular, evento tromboembólico ou sangramento maior e/ou não maior clinicamente relevante

Observamos uma taxa anual de 8,7% na ocorrência do desfecho composto por eventos tromboembólicos e hemorrágicos. Essa incidência foi ligeiramente superior às taxas encontradas no grupo varfarina dos estudos RE-LY e ENGAGE-AF, que foram de 7,64% e 8,11% ao ano, respectivamente.^26 , 27^ Os principais fatores independentes foram histórico de sangramento, TFT < 41% e diâmetro do AE > 44mm. Estes achados são comparáveis aos dados da literatura por representarem fatores de risco tanto para eventos tromboembólicos quanto para sangramentos.^36^ Dado semelhante foi encontrado por Kiliç et al., que avaliaram a eficácia e segurança da varfarina em clínicas da Turquia, sendo preditores independentes a história de sangramento e TFT < 50%.^36^

### Análise de pacientes com fibrilação atrial não valvar comparada com valvar

Ao analisarmos separadamente os grupos de FA valvar versus não valvar, aqueles com FA valvar eram mais jovens e com maior proporção de mulheres, em comparação com FA não valvar. Do total, 38,4% apresentavam doença reumática. Estes resultados revelam a prevalência elevada desta etiologia de valvopatia na população brasileira. De fato, a cardiopatia reumática permanece como problema relevante e negligenciado em muitos países em desenvolvimento, como observado no estudo *Rivaroxaban in Rheumatic Heart Disease–Associated Atrial Fibrillation* (INVICTUS).^37^

Em relação aos desfechos tromboembólicos no grupo FA valvar, ressaltamos a idade ≥ 59 anos como associada à menor ocorrência do desfecho composto e, ao contrário, o histórico de tromboembolismo, TFT < 38%, e TFG < 45 mL/min/m^2^ foram fatores de maior risco.

O grupo FA não valvar apresentou idade mais avançada e mais comorbidades, com pontuação dos escores de risco para tromboembolismo (CHADS_2_ e CHA_2_DS_2_-VASc) e sangramento (HAS-BLED) mais elevada em comparação com o grupo FA valvar, resultado oposto ao encontrado no Registro *Loire Valley Atrial Fibrillation Project* , onde os pacientes com valvopatia eram mais idosos, com CHA_2_DS_2_-VASc mais elevado, e com maior risco de eventos tromboembólicos do que pacientes sem valvopatia.^38^ A diferença dos nossos resultados em comparação com outros estudos possivelmente decorre da grande prevalência da cardiopatia reumática em nossa população, ao contrário do observado em países desenvolvidos, nos quais a etiologia da valvopatia é predominantemente degenerativa e calcífica.

### Forças e limitações do estudo

Destacamos os pontos fortes deste estudo, por representar a maior coorte e com maior duração de seguimento de pacientes com FA em uso de AVK no Brasil manejados na prática diária, portanto, expressam os resultados do mundo real, especificamente no contexto de sistema público de saúde. Outro aspecto positivo refere-se ao processo de adjudicação dos óbitos para determinação das causas de mortalidade, realizado de forma independente e seguindo padronização internacional e com base em estudos. Como possíveis limitações, este é um estudo observacional sem um grupo comparador como controle, portanto, apenas associações podem ser concluídas. Entretanto, um grupo controle não foi possível pelas barreiras do sistema público de saúde em termos de não disponibilidade de DOAC. Outro ponto refere-se ao fato de serem pacientes diagnosticados e anticoagulados em média por 10 anos, tornando esses pacientes usuários experientes de AVK.

## Conclusão

Nesta coorte identificamos que tromboembolismo ou sangramento prévios, TFG e TFT reduzidas e AE aumentado foram preditores independentes associados à ocorrência de desfechos clinicamente relevantes em pacientes com FA tratados com AVK.
