# Supplementary material for: Incidência e Preditores de Desfechos Clínicos em Pacientes com Fibrilação Atrial Valvar e Não Valvar em uso de Antagonistas da Vitamina K
Source: Arq Bras Cardiol. 2025 Jan 30;122(2):e20240147. [Article in Portuguese] doi: 10.36660/abc.20240147 (PMC11818470; doi:10.36660/abc.20240147)
Supplement: Translation

# Translation: Incidence and Predictors of Clinical Outcomes in Patients with Valvular and Nonvalvular Atrial Fibrillation Using Vitamin K Antagonists

**DOI:** 10.36660/abc.20240147i

**Authors:** Idelzuita Leandro Liporace, Gustavo Bernardes F. Oliveira, Lucas Bassolli de Oliveira Alves, Nadia Marchiori Galassi, Andreia Dias Jeronimo, Fernanda Maria Lopes, Gregory Y. H. Lip, Álvaro Avezum

**Affiliations:** 1 Brazil Instituto Dante Pazzanese de Cardiologia,São Paulo, SP – Brazil; 2 Brazil Hospital Alemão Oswaldo Cruz, São Paulo, SP – Brazil; 3 United Kingdom University of Liverpool – Liverpool Centre for Cardiovascular Science, Liverpool, Merseyside – United Kingdom; 4 United Kingdom Liverpool Heart & Chest Hospital, Liverpool – United Kingdom; 5 Denmark Aalborg Universitet – Aalborg Thrombosis Research Unit, Aalborg – Denmark

**Keywords:** Stroke, Thromboembolism, Hemorrhage, Atrial Fibrillation, Warfarin

## Abstract

**Background:**

Vitamin K antagonists (VKA) represent an important therapeutic strategy offered by the Brazilian Unified Public Health System to patients with atrial fibrillation (AF). However, predictors of relevant clinical outcomes are understudied in the real world.

**Objective:**

To determine the incidence and independent predictors of clinical outcomes in patients with valvular and nonvalvular AF treated with VKA.

**Methods:**

This prospective cohort included patients with valvular and nonvalvular AF receiving VKA for ≥ 1 year. The primary outcomes were cardiovascular death, thromboembolic events, and major and clinically relevant non-major bleeding, separately and as a composite outcome. The outcomes were independently adjudicated. P values < 0.05 were considered statistically significant.

**Results:**

The study included 1,350 patients, with a mean age of 69.2 (± 11.8) years, 53.6% female, followed up for 17 (15 - 19) months. The annual incidence of thromboembolic events and cardiovascular death was 4.4%, and predictors were prior thromboembolism (hazard ratio [HR] 2.12; 95% confidence interval [CI] 1.22 - 3.67), time in therapeutic range (TTR) < 50% (HR 1.98; 95% CI 1.16 - 3.37), and glomerular filtration rate (GFR) < 45 mL/min/1.73 m^2^(HR 2.76; 95% CI 4.82 - 1.58). The rate of major and clinically relevant non-major bleeding was 3.24% per year (95% CI 2.47 - 4.14), and predictors were prior bleeding (HR 2.60; 95% CI 1.47 - 4.61) and mechanical prosthesis (HR 1.91; 95% CI 1.15 - 3.15). The composite outcome was 8.7% per year, and predictors were prior bleeding (HR 1.70; 95% CI 1.07 - 2.70), TTR < 41% (HR 1.79; 95% CI 1.11 - 2.86), and left atrial diameter > 44 mm (HR 1.97; 95% CI 3.26 - 1.19).

**Conclusions:**

Prior thromboembolism or bleeding, reduced GFR and TTR levels, and enlarged left atrium were predictors of clinical outcomes in patients with AF treated with VKA.

## Introduction

Atrial fibrillation (AF) is the leading cardiac cause of thromboembolic stroke, accounting for increased rates of mortality, hospitalization, and disability.^1 , 2^Prevention of thromboembolic stroke is a priority in AF treatment, and studies in the 1990s revealed that vitamin K antagonists (VKA), compared with antiplatelet agents or placebo, reduced the risk of thromboembolic events by 64% and mortality by 26%.^3^Although treatment with VKA is highly effective in reducing the risk of stroke, there are difficulties in managing the medications due to several factors.^4^

Over the past 15 years, with the development of direct oral anticoagulants (DOAC), there has been a new perspective for the treatment of patients with AF. In fact, large pivotal studies with DOAC have demonstrated their non-inferiority when compared to VKA, and they are currently recommended as the anticoagulant therapy of choice for patients with AF in the absence of rheumatic mitral stenosis or mechanical prosthesis.^5 , 6^

However, in Brazil, DOAC have not been incorporated as antithrombotic therapy within the Unified Public Health System (SUS) due to their cost.^7^In our anticoagulation center, which is linked to the SUS, patients who would be indicated for the use of a DOAC are unable to afford the costs of the medication, and they continue to receive anticoagulation with VKAs.

Therefore, the object of our study was to identify the incidence of relevant clinical outcomes and independent predictors of cardiovascular death, thromboembolic events, major and clinically relevant non-major bleeding in patients with valvular and nonvalvular AF treated with VKA and monitored at an oral anticoagulation clinic in a large tertiary hospital specializing in cardiology.

## Methods

This was a prospective cohort study of patients aged ≥ 18 years, with valvular or nonvalvular AF or atrial flutter, treated with VKA (warfarin or phenprocoumon) for at least 1 year. Patients were recruited from July 2017 to July 2018 and followed from July 2017 to August 2019, in São Paulo, Brazil. The only exclusion criterion was pregnancy. According to the guidelines at the time of inclusion, patients were classified into the following 2 groups: a) valvular AF: moderate or severe mitral valve stenosis; mechanical or biological valve prostheses; or history of valve repair/plasty procedure; and b) nonvalvular AF: other cases without any of the aforementioned criteria. Baseline variables included demographic data, socioeconomic status, cardiovascular risk factors, relevant medical history, physical examination, and laboratory tests. Patients were anticoagulated according to individual weekly dose titration for the following therapeutic target international normalized ratio (INR) ranges: 2.5 to 3.5 (AF with mechanical valve prostheses) or 2 to 3 (all other patients with AF), as recommended by the guidelines. Follow-up was performed monthly after inclusion or earlier, according to clinical judgment. Time in therapeutic range (TTR) was calculated according to the Rosendaal method.^8^The CHADS_2_,^9^CHA_2_DS_2_-VASc,^10^HAS-BLED,^11^and SAMe-TT_2_R_2_^12^scores were calculated. To calculate the glomerular filtration rate (GFR), we used the Chronic Kidney Disease Epidemiology Collaboration (CKD-EPI) formula, and renal dysfunction was classified according to the National Kidney Foundation.^13^Alcohol abuse was considered as consumption of 8 or more drinks weekly, according to the National Institute on Alcohol Abuse and Alcoholism.^14^We used the classification of the International Society on Thrombosis and Haemostasis (ISTH), which defines bleeding as major (fatal, symptomatic in a critical area or organ, causing a fall in hemoglobin level of 2 g/dL or more, or leading to transfusion of 2 or more units of whole blood or red blood cells); clinically relevant non-major (acute or subacute clinically overt bleeding that does not meet the criteria for major bleeding but prompts a clinical response), and minor.^15^The causes of death were adjudicated by an independent observer, according to the International Classification of Diseases. In cases when there was no death certificate available or when death occurred at home or in another hospital, a verbal autopsy was performed to determine the cause of death.^16^At all consultations, patients were asked about major cardiovascular events, thromboembolism, and bleeding, which were classified and recorded on electronic forms according to severity, INR value at the time of the event, possible causes, and clinical course. The clinical outcomes were cardiovascular death, thromboembolic events, and major and clinically relevant non-major bleeding, separately and as a composite outcome.

This study was an investigator-initiated study, and it received approval from the institution’s Research Ethics Committee under CAAE number: 68007417.5.40.5462. All patients signed an informed consent form.

### Statistical analysis

The sample size was calculated according to studies on VKA with TTR ≥ 65% as an adequate indicator of anticoagulation. The type I error estimate was 5%, and the statistical power was 90%, resulting in a sample of 989 patients with AF to assess the quality of oral anticoagulation. Data normality was verified by inspection of histograms and application of the Shapiro-Wilk test. The characteristics of the valvular and nonvalvular groups were compared using Pearson’s chi-square test, unpaired Student’s t test, or Mann-Whitney test, when applicable.

Continuous variables were expressed as mean ± standard deviation or median and interquartile range (IQR) according to data normality, and categorical variables were described as absolute (n) and relative (%) frequencies. The frequency of outcomes and events of interest was described as absolute number, incidence rate, and annualized incidence rate per 100 person-years. The cumulative incidence of outcomes during the study was estimated using the Kaplan-Meier method. The prognostic value of potential predictors of outcomes was quantified by hazard ratios, which were estimated using Cox models. Multivariate analyses were used to define the set of risk factors for the occurrence of outcomes, including all variables selected in the univariate analyses with significance level of 10% and variables with clinical relevance.^17^The assumption of proportionality of risks was assessed by analyzing Schoenfeld residuals, and hazard ratios and 95% confidence intervals derived from the Cox method were reported. The final models were adjusted for age, sex, presence of valvular disease, polypharmacy, and history of cancer, because they are considered clinically relevant variables for the outcomes. The discriminative accuracy of the final models was assessed by the area under the receiver operating characteristic curve of the values predicted by the models (C index). The results were presented for the overall population and stratified according to type of AF (valvular or nonvalvular). The cutoff points for continuous variables were established according to clinical criteria or statistical criteria using maximally selected rank statistics (maxstat).^18^Missing echocardiographic data were imputed using multiple imputation by chained equations (MICE), based on age, sex, presence of valvular disease, and use of mechanical prosthesis.^19^All statistical tests were 2-sided with p values < 0.05 denoting statistical significance. All analyses were performed using RStudio 1.3.959 statistical software.^20^

Cox proportional hazards models were used for multivariate adjustment of logistic regression for the study outcomes, and Wald statistics were applied for hypothesis testing.

## Results

We recruited 1411 patients with AF, and 61 were excluded during follow-up, as illustrated in Figure 1 . Regarding overall description, mean age was 69.2 (± 11.8) years, and women accounted for 53.6%. Warfarin represented 77.7% of VKA. The mean time of VKA use before inclusion was 10.4 years. According to clinical definition, 52.8% were considered nonvalvular AF, 70% permanent AF, and 8.5% atrial flutter. The prevalence of comorbidities was elevated among patients in this sample, as displayed in Table 1 .

**Figure 1:** – Study flowchart.

**Table 1:** – Clinical characteristics of all patients with atrial fibrillation and comparison between patients with nonvalvular and valvular atrial fibrillation

		Etiology of AF		
Variables	Total sample (1350)	Nonvalvular (n = 713)	Valvular (n = 637)	p
Age, mean (SD)	69.2 (±11.8)	73.7 (±10.0)	64.1 (±11.5)	< 0.001
Age > 60 years, n (%)	1.084 (79.5)	657 (92.1)	427 (67.0)	< 0.001
Female sex, n (%)	724 (53.6)	314 (44.0)	410 (64.4)	< 0.001
White/Asian ethnicity, n (%)*	845 (62.7)	448 (62.9)	397 (62.5)	0.879
Elementary school, n (%)*	980 (73.0)	557 (78.5)	517 (81.7)	0.141
Arterial hypertension, n (%)	1.011 (74.9)	610 (85.6)	401 (63.0)	< 0.001
Dyslipidemia, n (%)	761 (56.4)	495 (69.4)	266 (41.8)	< 0.001
Diabetes mellitus, n (%)	351 (26.0)	248 (34.8)	103 (16.2)	< 0.001
Coronary artery disease, n (%)	234 (17.3)	177 (24.8)	57 (8.9)	< 0.001
Renal function, n (%)*				< 0.001
GFR ≥ 90 mL/min/m^2^	231 (17.1)	68 (9.6)	163 (25.6)	
GFR 60 to 89 mL/min/m^2^	637 (47.2)	336 (47.2)	301 (47.3)	
GFR 45 to 59 mL/min/m^2^	277 (20.5)	175 (24.6)	102 (16.0)	
GFR 30 to 44 mL/min/m^2^	159 (11.8)	104 (14.6)	55 (8.6)	
GFR < 30 mL/min/m^2^	44 (3.3)	29 (4.1)	15 (2.4)	
Ejection fraction, %, median (q1-q3)	59 (18; 79)	60 (49-64)	58 (49-62)	0.297
LA diameter, median (q1-q3)	49 (24; 126)	47 (42-52)	52 (48-58)	< 0.001
Paroxysmal AF, n (%)	314 (23.3)	185 (25.9)	129 (20.3)	0.013
Atrial flutter	115 (8.5)	64 (9.0)	51 (8.0)	0.524
ACO time, median (q1-q3)	10.4 (7.7-14.7)	119 (97-154)	140 (73-215)	< 0.001
Rosendaal TTR < 65%	664 (49.2)	290 (40.7)	374 (58.7)	< 0.001
Antiplatelet therapy, n (%)	131 (9.7)	90 (12.6)	41 (6.4)	< 0.001
Phenprocoumon-warfarin switch, n (%)	301 (22.3)	129 (18.1)	172 (27.0)	< 0.001
Polypharmacy, n (%)	789 (58.4)	484 (67.9)	305 (47.9)	< 0.001
Heart failure, n (%)	493 (36.5)	250 (35.1)	243 (38.1)	0.240
BMI ≥ 30 kg/m^2^*	371 (27.5)	228 (32.0)	143 (22.4)	< 0.001
Prior ischemic stroke, (%)	218 (16.1)	117 (16.4)	101 (15.9)	0.782
Prior thromboembolism, n (%)	263 (19.5)	132 (18.5)	131 (20.6)	0.342
Cancer, n (%)	32 (2.4)	25 (3.5)	7 (1.1)	0.004
Altered liver function, n (%)	22 (1.6)	13 (1.8)	9 (1.4)	0.552
Prior bleeding, n (%)	164 (12.1)	79 (11.1)	85 (13.3)	0.204
CHADS2, median (q1-q3)	3.0 (2.0 – 4.0)	3.0 (2.0 - 4.0)	2.0 (1.0 - 3.0)	< 0.001
CHA2DS2-VASC, median (q1-q3)	3.0 (2.0 – 5.0)	4.0 (3.0 - 5.0)	3.0 (2.0 - 4.0)	< 0.001
HAS-BLED, median (q1-q3)	3.0 (2.0 – 3.0)	3.0 (3.0 - 4.0)	3.0 (2.0 - 3.0)	< 0.001
SAMe-TT2R2, median (q1-q3)	1.0 (1.0 – 3.0)	2.0 (1.0 - 3.0)	2.0 (1.0 - 3.0)	0.094
**Event rate**				
Composite outcome: bleeding + TE event, n (%)	118 (8.7)	57 (8.0)	61 (9.6)	0.304
Composite outcome: TE event + CV death (%)	60 (4.4)	31 (4.3)	29 (4.6)	0.855
TE event, n (%)	23 (1.7)	10 (1.4)	13 (2.0)	0.366
Composite outcome: major + CRNM bleeding, n (%)	62 (4.6)	26 (3.6)	36 (5.7)	0.079
CV death	42 (3.1)	25 (3.5)	17 (2.7)	0.565

*AF: atrial fibrillation; ACO: anticoagulant; BMI: body mass index; CKD-EPI: Chronic Kidney Disease Epidemiology Collaboration; CRNM: clinically relevant non-major; CV: cardiovascular; GFR: glomerular filtration rate; HR: hazard ratio; LA: left atrial; q1-q3: interquartile ranges; SD: standard deviation; TTR: time in therapeutic range.*

### Clinical outcomes

Patients were followed for a median of 17 (IQR 15 to 19) months. All-cause mortality occurred in 6.2%, and 50% of these cases were due to cardiovascular causes, with an overall 2-year survival rate of 90.5%. Ischemic or thrombotic events occurred in 1.7%, with an annual rate of 1.18. Major and clinically relevant non-major bleeding events were 4.6%, with an annual rate of 3.23, as displayed in Table 1 .

### Composite outcome of cardiovascular death or thromboembolic event

The cumulative incidence of the outcome was 4.4% per year and 7.4% at 2 years ( Figure 2 ). Multivariate analysis identified prior thromboembolism, GFR < 45 mL/min/m^2^, TTR < 50%, and left atrial (LA) diameter as predictors independently associated with the occurrence of the composite outcome of cardiovascular death or thromboembolic event, as displayed in Table 2 .

**Figure 2:** – Cumulative incidence of the composite outcome of cardiovascular death and thromboembolic events.

**Table 2:** – Multivariate analysis of predictors associated with clinical thromboembolic outcomes (A), bleeding outcomes (B), and composite thromboembolic and bleeding outcomes (C), in the total population with atrial fibrillation

**A**				
	Hazard ratio (95% CI) *	**p**	**Adjusted hazard ratio (95% CI)^ⱡ^**	**p**
TTR < 50%	1.98 (1.16-3.37)	0.013	1.95 (1.12-3.40)	0.019
GFR < 45 mL/min/m^2^	2.76 (1.58-4.82)	< 0.001	2.62 (1.43-4.82)	0.002
Prior thromboembolism	2.12 (1.22-3.67)	0.015	2.08 (1.21-3.61)	0.009
LA diameter (continuous)	1.03 (1.00-1.05)	0.015	1.03 (1.01-1.05)	0.009
* n = 1348; C index = 0.714 (± 0.03). CI: confidence interval; GFR: glomerular filtration rate; LA: left atrium; TTR: time in therapeutic range.^ⱡ^Adjusted for: age groups (60 to 80 years, < 60 years, and > 80 years), sex, valvular atrial fibrillation, polypharmacy, and neoplasia. C index = 0.717 (± 0.03).				
**B**				
	Hazard ratio (95% CI) *	**p**	**Adjusted hazard ratio (95% CI)^ⱡ^**	**p**
Mechanical prosthesis	1.91 (1.15-3.15)	0.012	1.98 (0.89-4.40)	0.094
Prior bleeding	2.60 (1.47-4.61)	0.001	2.51 (1.41-4.47)	0.002
* n = 1,350; C index = 0.634 (± 0.03). CI: confidence interval.^ⱡ^Adjusted for: age groups (60 to 80 years, < 60 years, and > 80 years), sex, valvular atrial fibrillation, polypharmacy, and cancer. C index = 0.649 (± 0.03).				
**C**				
	Hazard ratio (95% CI) *	**p**	**Adjusted hazard ratio (95% CI)^ⱡ^**	**p**
Rosendaal TTR < 41%	1.79 (1.11-2.86)	0.016	1.74 (1.08-2.82)	0.024
Left atrium > 44 mm	1.97 (1.19-3.26)	0.008	1.93 (1.14-3.24)	0.014
History of bleeding	1.70 (1.07-2.70)	0.026	1.66 (1.04-2.65)	0.033

* n = 1350; C index = 0.622 (± 0.02). CI: confidence interval; TTR: time in therapeutic range.^ⱡ^Adjusted for: age groups (60 to 80 years, < 60 years, and > 80 years), sex, valvular atrial fibrillation, polypharmacy, cancer, and prior thromboembolism. n = 1350; C index = 0.638 (± 0.02). Cox proportional hazards models were used for multivariate risk adjustment of the study outcomes, and Wald statistics were applied for hypothesis testing.

### Composite outcome of major and/or clinically relevant non-major bleeding

The annual bleeding rate was 3.23%, and patients with a mechanical valve prosthesis and those with prior bleeding had a higher risk of new bleeding ( Table 2 and Figure 3 ).

**Figure 3:** – Cumulative incidence of the composite outcome of major and clinically relevant non-major bleeding.

### Composite outcome of cardiovascular death, thromboembolic event, or major and/or clinically relevant non-major bleeding

A combination of thrombotic and bleeding outcomes was observed in 8.7%. Figure 4 shows the cumulative incidence at 2 years. The independent predictors were prior bleeding, TTR < 41%, and LA diameter > 44 mm, as displayed in Table 2 .

**Figure 4:** – Cumulative incidence of the composite outcome of cardiovascular death, thromboembolic, and bleeding events.

### Comparison between patients with nonvalvular versus valvular AF

Of the total sample, 52.8% were classified as nonvalvular AF and 47.2% as valvular AF ( Figure 1 ). The characteristics of patients with nonvalvular and valvular AF showed a statistically significant difference in various aspects, leading to the analysis of outcomes according to both groups. The mean age of patients with nonvalvular AF was 73.7 years versus 64.1 years in patients with valvular AF. At the time of inclusion, 92.1% of patients with nonvalvular AF were over 60 years old, compared with 67% in the valvular AF group. Women represented 64.4% of the valvular AF group versus 44% of patients with nonvalvular AF. Regarding self-reported ethnicity and education, no differences were observed between the groups ( Table 1 ).

Patients with nonvalvular AF had a higher prevalence of systemic arterial hypertension, dyslipidemia, diabetes mellitus, obesity, coronary artery disease, severe renal failure, use of antiplatelet agents, and polypharmacy. Permanent AF was also more frequent among those with valvular AF, while paroxysmal AF was more prevalent in nonvalvular AF. The CHADS_2_, CHA_2_DS_2_-VASc, and HAS-BLED risk scores were higher in patients with nonvalvular AF versus valvular AF. The median SAMe-TT_2_R_2_score was similar for both groups. Longer duration of anticoagulant use and patients with lower TTR (< 65%) were more frequent in the valvular AF group, as displayed in Table 1 .

Among patients with nonvalvular AF, TTR < 50%, LA diameter, and GFR < 45 mL/min/m2 (hazard ratio 2.20; 95% confidence interval 1.01 to 4.77) were independent predictors for the composite outcome of ischemic and bleeding events. TTR < 50%, LA diameter, and prior bleeding were independent predictors for the occurrence of the composite outcome of thrombotic and bleeding events, as displayed in Table 3 . Isolated and composite outcomes in the valvular AF group are also shown in Table 4 .

**Table 3:** – Multivariate analysis of predictors associated with thromboembolic outcomes (A) and composite thromboembolic and bleeding outcomes (B), in the nonvalvular AF group

**A**				
	Hazard ratio (95% CI) *		**p**	
Rosendaal TTR < 50%	4.12 (1.97-8.63)		< 0.001	
GFR < 45 mL/min/m^2^	2.20 (1.01-4.77)		0.046	
LA diameter (continuous)	1.05 (1.02-1.07)		< 0.001	
* n = 712; C index = 0.755 (± 0.05). CI: confidence interval; GFR: glomerular filtration rate; LA: left atrium; TTR: time in therapeutic range. Model stratified by age ≥ 84 years. This variable, although significant, violated the assumption of proportionality of risks.				
**B**				
	Hazard ratio (95% CI) *	**p**	**Adjusted hazard ratio (95% CI)^ⱡ^**	**p**
TFT Rosendaal < 50%	2.54 (1.42-4.54)	0.002	2.64 (1.47-4.76)	0.001
Sangramento prévio	2.25 (1.21-4.17)	0.010	2.18 (1.17-4.07)	0.015
Diâmetro AE (contínuo)	1.03 (1.01-1.05)	0.001	1.04 (1.02-1.06)	0.001

* n = 713; C index = 0.673 (± 0.04). CI: confidence interval; LA: left atrium; TTR: time in therapeutic range.^ⱡ^Adjusted for: age, sex, polypharmacy, and neoplasia. C index = 0.671 (± 0.04). Cox proportional hazards models were used for multivariate risk adjustment of the study outcomes, and Wald statistics were applied for hypothesis testing.

**Table 4:** – Multivariate analysis of predictors associated with thromboembolic outcomes (A) and composite thromboembolic and bleeding outcomes (B), in the valvular AF group

**A**				
	Hazard ratio (95% CI) *		**p**	
Age ≥ 59 years	0.38 (0.18-0.81)		0.013	
GFR < 45 mL/min/m^2^	2.84 (1.11-7.25)		0.029	
* n = 636; C index = 0.629 (± 0.05). CI: confidence interval; GFR: glomerular filtration rate.				
**B**				
	Hazard ratio (95% CI) *	**p**	**Adjusted hazard ratio (95% CI)^ⱡ^**	**p**
Rosendaal TTR < 38%	2.08 (1.10-3.92)	0.024	1.96 (1.02-3.74)	0.042
Prior thromboembolism	1.73 (1.00-3.01)	0.052	1.78 (1.02-3.11)	0.044
Diabetes mellitus	1.83 (1.03-3.25)	0.040	1.71 (0.93-3.13)	0.083
GFR < 45 mL/min/m^2^	1.93 (1.00-3.73)	0.050	2.09 (1.04-4.19)	0.039

* n = 636; C index = 0.649 (± 0.03). CI: confidence interval; GFR: glomerular filtration rate; TTR: time in therapeutic range.^ⱡ^Adjusted for: age, sex, polypharmacy, and neoplasia. C index = 0.659 (± 0.03). ox proportional hazards models were used for multivariate risk adjustment of the study outcomes, and Wald statistics were applied for hypothesis testing.

## Discussion

As of the date this manuscript was submitted, to the best of our knowledge, this represents the largest Brazilian prospective cohort derived from the real world with experience using VKA in patients with AF in a broad spectrum. We evaluated predictors of relevant clinical outcomes, namely, thrombotic events, bleeding, and deaths in patients with AF or atrial flutter, considered valvular or nonvalvular, treated in a public hospital, which reflects the most common clinical practice in this condition. We highlight the demographic characteristics of advanced age, predominance of women, and low economic and educational levels.

During the follow-up of 1950 person-years, we observed an elevated prevalence of comorbidities, mainly hypertension, heart failure, diabetes mellitus, obesity, and renal dysfunction, which correspond to risk factors related to the development of AF.^21^The median TTR observed in our sample was 65%, lower than values observed in Spain (70.3%) and Germany (81.4%),^22^but similar to the rate found in the United States and Canada (64.1%)^23^and greater than the rates revealed in Lithuania (40%)^24^and African countries (30.8%).^25^

### Composite outcome of cardiovascular death or thromboembolic event

The occurrence of the composite outcome of cardiovascular death or thromboembolic event was observed in 4.4%, similar to the annualized rate of the efficacy outcome found in patients using warfarin in the randomized study Edoxaban versus Warfarin in patients with Atrial Fibrillation (ENGAGE-AF), which was 4.43%.^26^However, it was slightly higher than the rate of 3.51% found in the clinical trial Dabigatran versus warfarin in patients with atrial fibrillation (RE-LY) and the rate of 2.2% in patients from the study Rivaroxaban versus warfarin in nonvalvular atrial fibrillation (ROCKET-AF).^27 , 28^This difference may be related to the presence of patients with greater clinical severity, mechanical prostheses, and moderate to severe mitral stenosis in our sample. The total mortality rate during study follow-up was 6.2%, which was higher than the rates found in the warfarin groups in large pivotal studies using DOAC. In the RE-LY clinical trial, the overall mortality rate was 4.13%; in the ROCKET-AF trial, it was 4.9%, and in the Apixaban versus warfarin in patients with atrial fibrillation (ARISTOTLE) trial, it was 3.94%.^29^In relation to cardiovascular mortality, we observed 3.1%, similar to the ENGAGE-AF clinical trial, which found 3.17%, but slightly higher than the rate observed in the ARISTOTLE trial, namely, 2.02%.^26 , 29^Our hypothesis for the results is based on the severity of the patients treated at our anticoagulation clinic, with a large volume of cases referred in a more advanced stage of heart disease, with mechanical prostheses or moderate to severe mitral stenosis, unlike those selected for randomized clinical trials. The annual incidence of thromboembolic events was 1.18%, which was lower than the incidences observed in the warfarin groups of the RE-LY (1.69%), ROCKET-AF (2.2%), ARISTOTLE (1.27%), and ENGAGE-AF (1.5%) trials, as well as the study that compared warfarin with acetylsalicylic acid or placebo, with a 1.4% incidence of thromboembolic events.^26 - 30^

Independent risk factors for the occurrence of the composite outcome of cardiovascular death or thromboembolic events were prior thromboembolism, TTR < 50%, and GFR < 45 mL/min/m^2^. Similar results were observed in the Global Anticoagulant Registry in the FIELD–Atrial Fibrillation (GARFIELD-AF), in which prior ischemic stroke or transient ischemic attack were also associated with a significantly higher risk of total mortality. In the same registry, patients using warfarin with TTR < 65% had a 2.6-fold increased risk of ischemic stroke and a 2.4-fold increased risk of total mortality, compared to patients with TTR levels that were considered adequate.^31^In a study published by Jones et al., a 10% reduction in TTR was associated with a 29% increase in risk of mortality and an increase of 10% to 12% in thromboembolic events, including ischemic stroke,^32^which was in agreement with our results.

### Composite outcome of major and/or clinically relevant non-major bleeding

In relation to the composite outcome of major and/or clinically relevant non-major bleeding, according to the ISTH criteria, we observed an annual incidence of 3.24%, lower than the rates demonstrated in the warfarin groups of the ENGAGE-AF and ROCKET-AF studies, 13.02% and 14.5%, respectively.^26 , 28^The findings of the present study were similar to the rates found in a systematic review of randomized studies on warfarin compared to those without an anticoagulant, with average annual frequencies of 3% for major bleeding and 9.6% for major and minor bleeding.^26^

The predictors associated with the occurrence of the composite outcome of bleeding were the presence of mechanical valve prostheses and prior bleeding. This result was similar to the one described by Priksri et al., with the presence of a mechanical prosthesis in the mitral position being an independent risk factor for the occurrence of warfarin-related bleeding.^33^

The annual rate of major bleeding alone in this study was 1.29%, which was lower than the rates observed in the groups using warfarin in the ROCKET-AF, ARISTOTLE, and ENGAGE-AF studies, 3.4%, 3.09%, and 3.43% respectively.^26 , 28 , 29^

In our study, severe renal dysfunction was associated with the occurrence of thromboembolic events, but it was not associated with an increase in major or clinically relevant bleeding, contrary to what was observed in other studies.^34^For example, Lip et al. evaluated a cohort of 7,329 patients with AF from the Stroke Prevention Using an ORal Thrombin Inhibitor in Atrial Fibrillation (SPORTIF III and V) studies, which compared the use of warfarin with ximelagatran, and the presence of renal failure (GFR < 50 mL/min/m^2^) was one of the factors associated with the occurrence of bleeding.^35^

### Composite outcome of cardiovascular death, thromboembolic event, or major and/or clinically relevant non-major bleeding

We observed an annual rate of 8.7% in the occurrence of the composite outcome of thromboembolic and bleeding events. This incidence was slightly higher than the rates found in the warfarin groups of the RE-LY and ENGAGE-AF studies, 7.64% and 8.11% per year, respectively.^26 , 27^The main independent factors were history of bleeding, TTR < 41%, and LA diameter > 44 mm. These findings are comparable to data from the literature because they represent risk factors for both thromboembolic events and bleeding.^36^Similar data were found by Kiliç et al., who evaluated the efficacy and safety of warfarin in clinics in Turkey and found that history of bleeding and TTR < 50% were independent predictors.^36^

### Analysis of patients with nonvalvular versus valvular atrial fibrillation

When analyzing the valvular versus nonvalvular AF groups separately, patients with valvular AF were younger, and there was a higher proportion of women, compared to nonvalvular AF. Of the total, 38.4% had rheumatic disease. These results reveal the high prevalence of this etiology of valvular heart disease in the Brazilian population. In fact, rheumatic heart disease remains a relevant and neglected issue in many developing countries, as observed in the Rivaroxaban in Rheumatic Heart Disease–Associated Atrial Fibrillation (INVICTUS) study.^37^

In relation to thromboembolic outcomes in the valvular AF group, we highlight age ≥ 59 years as associated with a lower occurrence of the composite outcome; on the other hand, history of thromboembolism, TTR < 38%, and GFR < 45 mL/min/m^2^were greater risk factors.

The nonvalvular AF group was older and had more comorbidities, with higher risk scores for thromboembolism (CHADS_2_and CHA_2_DS_2_-VASc) and bleeding (HAS-BLED), compared with the valvular AF group. These results are the opposite of those found in the registry of the Loire Valley Atrial Fibrillation Project, where patients with valvular heart disease were older, had higher CHA_2_DS_2_-VASc, and showed a higher risk of thromboembolic events than patients without valvular heart disease.^38^The difference in our results compared with other studies is possibly due to the high prevalence of rheumatic heart disease in our population, contrary to what is observed in developed countries, where the etiology of valvular heart disease is predominantly degenerative and calcific.

### Study strengths and limitations

We highlight this study’s strengths, as it represents the longest follow-up period and the largest cohort of patients with AF using VKA in Brazil managed in daily practice; therefore, they reveal real-world results, specifically in the context of the Brazilian public health system. Another positive aspect is related to the process of adjudication of deaths to determine the causes of mortality, which was carried out independently, following international standardization and based on studies. Regarding possible limitations, this was an observational study without a comparator group as a control; therefore, only associations can be concluded. However, a control group was not possible due to the barriers of the public health system in terms of non-availability of DOAC. Another issue is related to the fact that patients were diagnosed and anticoagulated for an average of 10 years, making them experienced users of VKA.

## Conclusion

In this cohort, we identified prior thromboembolism or bleeding, reduced GFR and TTR, and LA enlargement as independent predictors associated with the occurrence of clinically relevant outcomes in patients with AF treated with VKA.
